# Carcinoma Breast-Like Giant Complex Fibroadenoma: A Clinical Masquerade

**DOI:** 10.7759/cureus.12611

**Published:** 2021-01-10

**Authors:** Jitendra S Nigam, Prerna Tewari, Tanya Prasad, Tarun Kumar, Anil Kumar

**Affiliations:** 1 Pathology/Lab Medicine, All India Institute of Medical Sciences, Patna, IND; 2 Surgery, All India Institute of Medical Sciences, Patna, IND

**Keywords:** breast, fibroadenoma, carcinoma, cyst, calcifications

## Abstract

Fibroadenoma (FA) is a benign, painless, solid breast tumor that commonly occurs in young adult females. The term complex FA is used when it is associated with any of the following: cyst >3 mm, epithelial calcifications, sclerosing adenosis, or papillary apocrine metaplasia. FAs of size more than 5 cm or weighing more than 500 g are considered as giant FAs. Giant FAs are rare, and because of hormonal sensitivity, they commonly occur in pregnant or lactating women. We report the case of a 26-year-old female with a large breast mass that was clinically as well as grossly masquerading as breast carcinoma and turned out to be a giant complex FA on microscopy.

## Introduction

Fibroadenoma (FA) is a benign, painless, solid breast tumor that commonly occurs in young adult females with peak incidence between 14 to 35 years of age but can be found in any age group [1-3]. The epithelial components of FA are affected by many elements, including estrogen, progesterone, pregnancy, and lactation [1-3]. Due to its sensitivity to estrogen and progesterone, FA increases in size during pregnancy and tends to show atrophic changes during menopause [1-3]. FA patients have a higher relative risk (1.60-2.17) of developing breast carcinoma compared to women without FA [1-3]. The term complex FA is used when it is associated with any of the following: cyst >3 mm, epithelial calcifications, sclerosing adenosis, or papillary apocrine metaplasia [1-3]. Patients with complex FA have a higher relative risk (2.27-3.10) risk of developing breast carcinoma than patients with FA without complex features [3]. Patients usually present with firm-to-hard, painless, rubbery, mobile nodules of variable size with well-defined borders [1,4]. FAs measuring more than 5 cm or weighing more than 500 g are considered giant FAs, which account for approximately 0.5% to 2% of the FAs [4]. We report the case of a 26-year-old female with a large breast mass that was clinically and grossly masquerading as breast carcinoma.

## Case presentation

**Figure 1:**
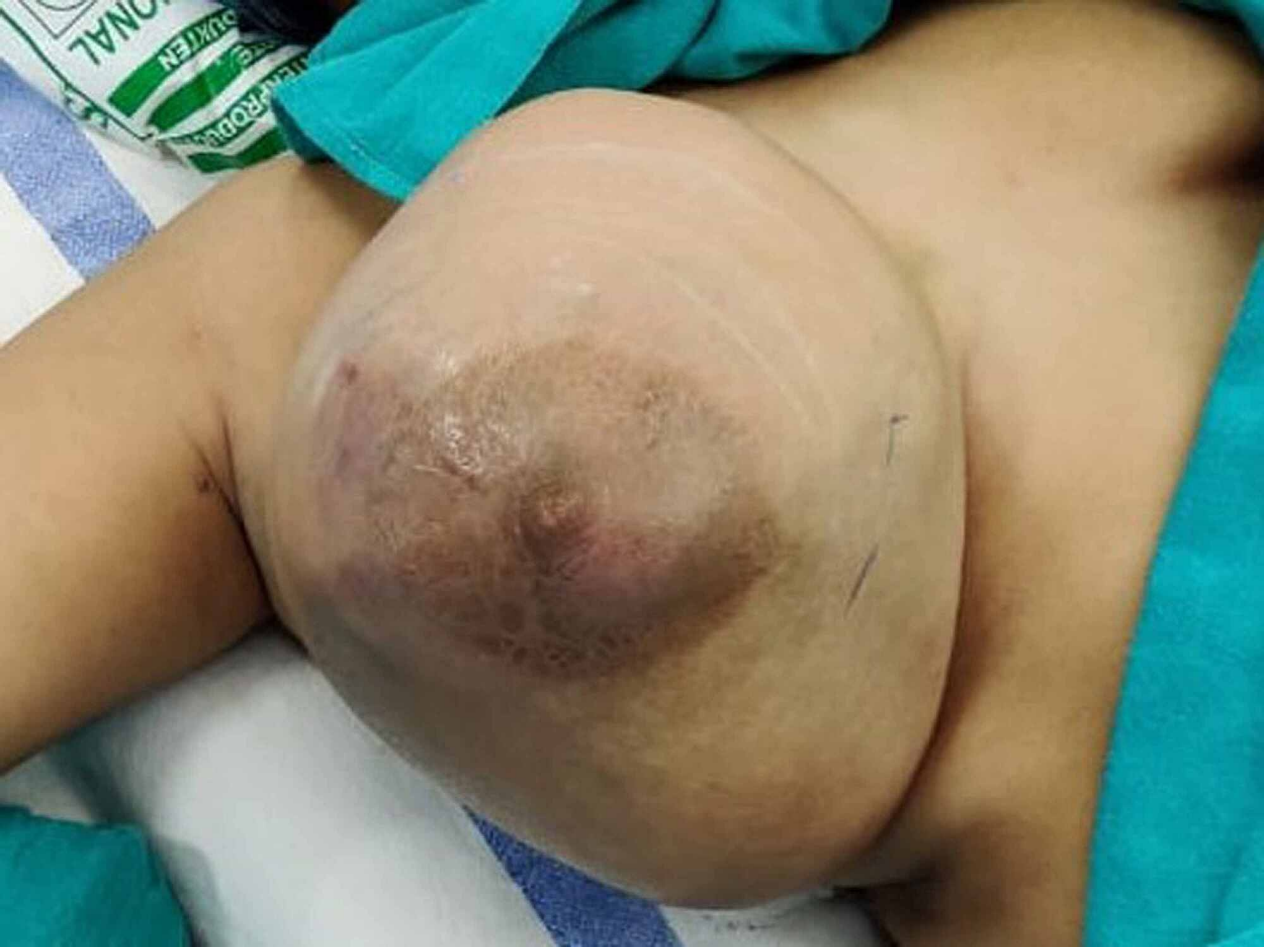
Preoperative image of the large lesion involving the entire breast.

A 26-year-old female presented to the surgical outpatient department with a unilateral right-sided breast lump for one year. The swelling was associated with pain. The patient was not pregnant and not lactating. Physical examination revealed a very large right breast lump of approximately 25 × 15 cm involving the whole breast. The overlying skin was mainly unremarkable with some redness at the 9'o clock position. No nipple retraction or puckering of the skin was identified (Figure 1).

**Figure 2:**
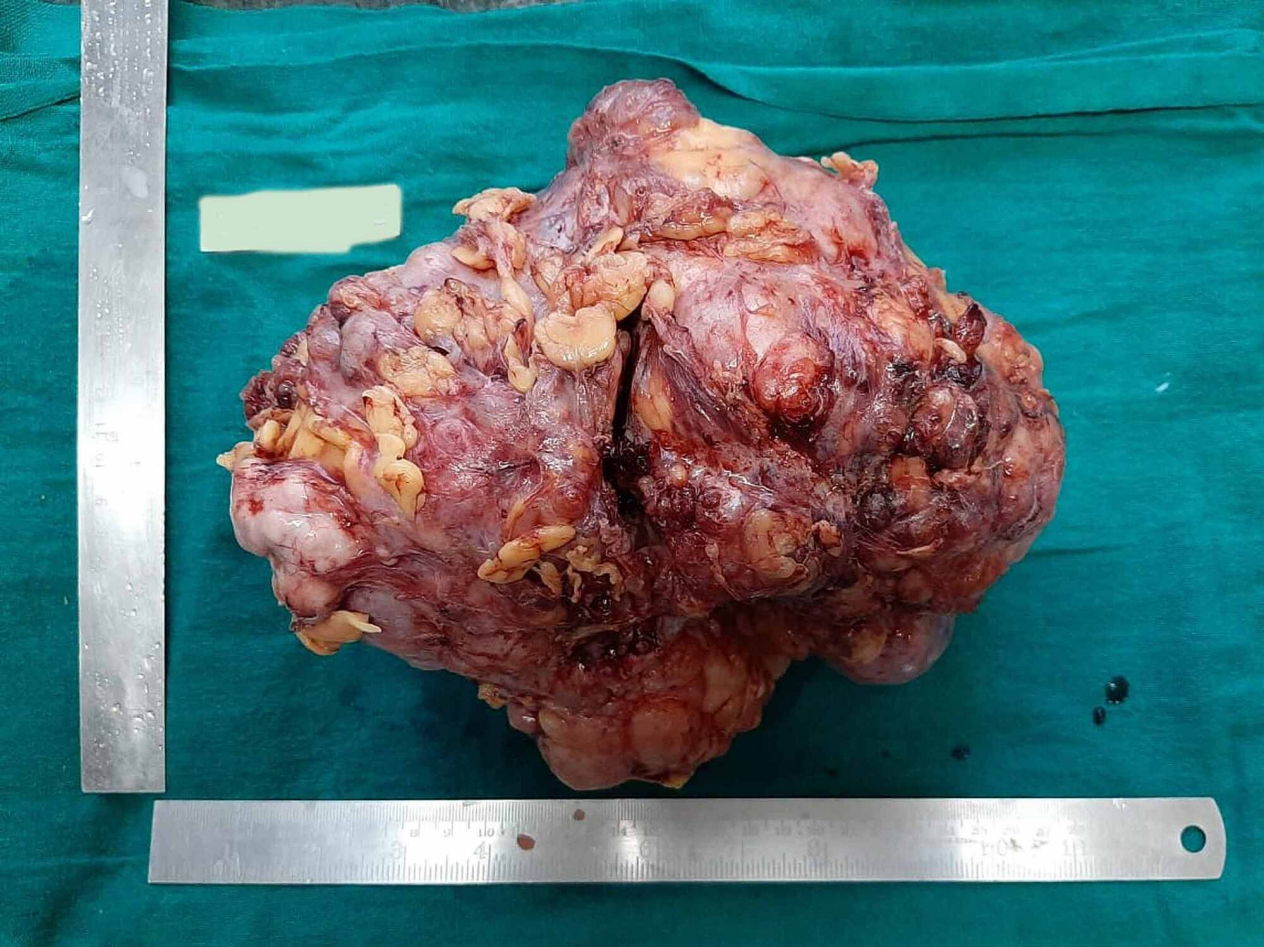
Gross Large, bosselated mass with the presence of multiple nodules on the external surface.

Axillary lymphadenopathy was not noted. True-cut biopsy showed predominantly fibrocollagenous tissue along with few benign ductal elements. No increase in stromal cellularity, atypia, mitosis, or malignancy was noted. Complete surgical excision of the breast lump was done and sent for histopathological examination. The lumpectomy specimen was partially covered by skin ellipse without nipple, globular, firm nodular tissue mass measuring 23 × 14 × 7 cm. The outer surface of the specimen was bosselated due to the presence of multiple nodules (Figure 2).

**Figure 3:**
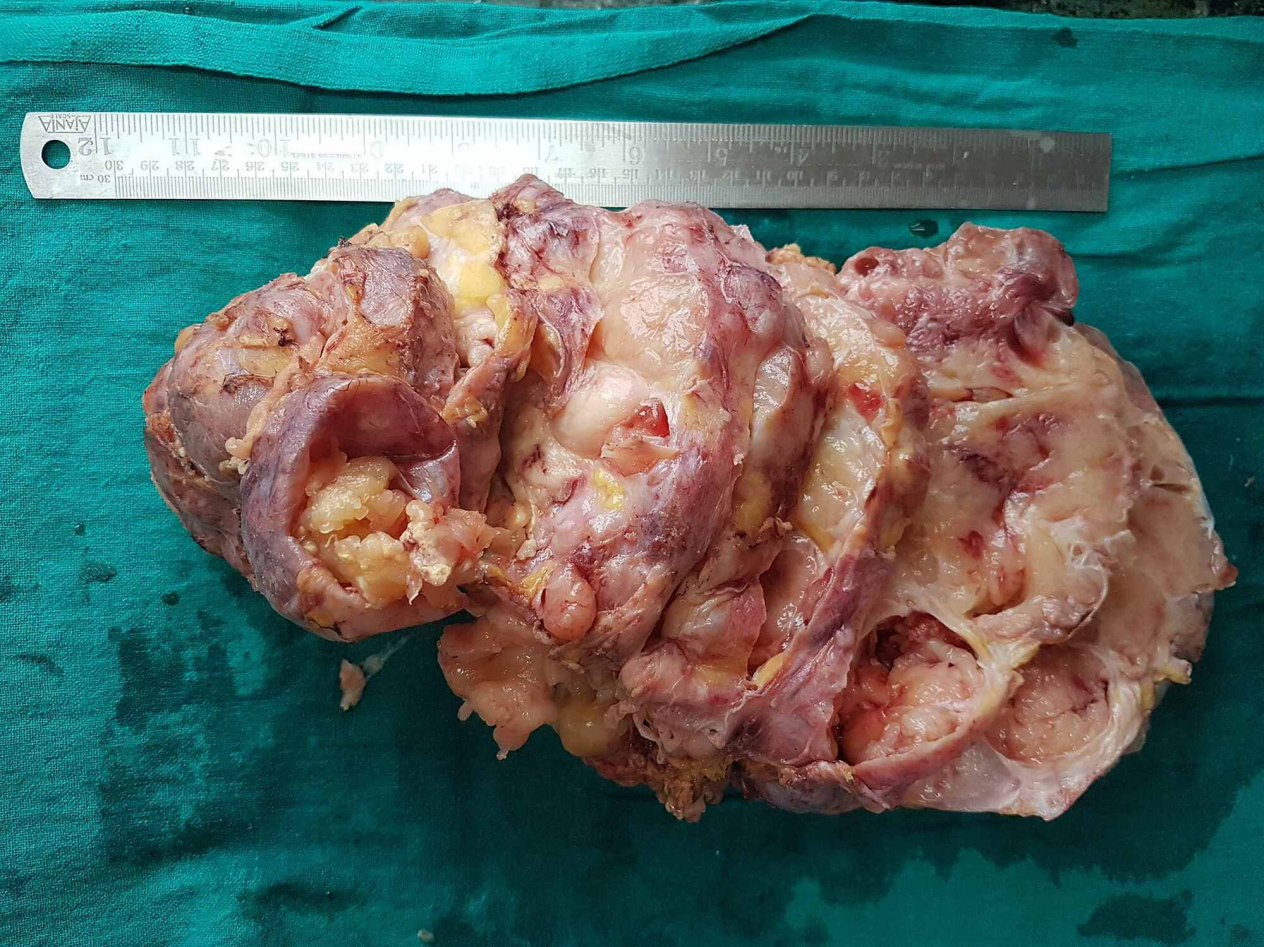
Cut surface showing multiple cystic areas filled with mucoid material and gray-white to grayish yellow solid areas.

The serial sectioning revealed multiple cysts measuring (0.5-4.2 cm) filled with mucoid material and intervening tan-yellow solid areas (Figure 3).

**Figure 4:**
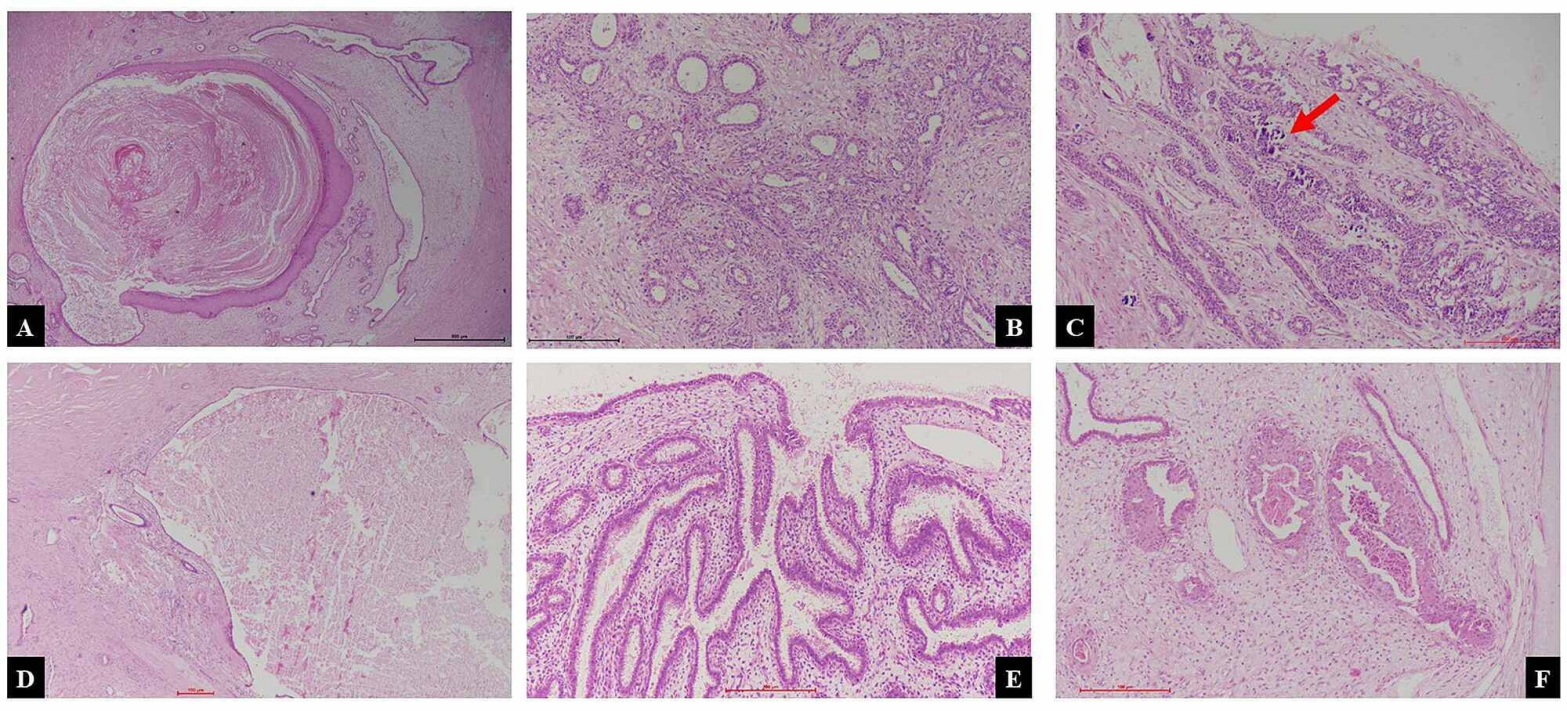
(A) Cyst with squamous metaplasia and features of inclusion cyst (H&E 20×). (B) Area of sclerosing adenosis (H&E 100×). (C) Adenosis with calcification (red arrow) (H&E 100×). (D) Cyst lined by flattened epithelium filled with secretion (H&E 40×). (E) Papillary apocrine metaplasia (H&E 100×). (F) Ductal squamous metaplasia (H&E 100×). H&E, hematoxylin and eosin

The gross differentials were mucinous carcinoma or malignant phyllodes. Histopathological examination showed epithelial and stromal proliferation with some cystic dilatation of ducts and focal apocrine changes. Some sections also showed areas of sclerosing adenosis, foci of microcalcification, and stromal hyalinization. Focal squamous metaplasia was also seen (Figure 4).

No atypia or dysplasia was noted in multiple levels. Based on tumor size and complex features, the final diagnosis of giant complex FA was given. The 12-month follow-up of the patient was uneventful and no recurrence was noted.

## Discussion

FAs are common benign breast lesions comprising both epithelial and stromal elements [1,2,4]. They generally occur as a single breast mass called a breast mouse due to high mobility within the breast parenchyma [1,2,4]. Giant FAs are rare and commonly occur in pregnant or lactating women as epithelial and stromal elements are sensitive to estrogen, progesterone, and prolactin [1,2,4]. Complex features may be seen in any size of FAs [2]. Patient age for complex FAs ranges from 18 to 70 years and are common in older patients [2,5,6]. It has been reported that 15.7% to 40.4% of all FAs are complex [2,5-7]. Some studies have reported that no clinical and radiologic features can help discriminate complex FAs from simple FAs [7]. However, some studies observed that on sonography, there are aggressive features such as noncircumscribed margins, complex echo structure, and posterior acoustic enhancement that are more common in complex FAs [5,6]. Lee et al. observed that nearly 50% of complex FAs belonged to Breast Imaging Reporting and Data System lexicon category 4 and show significantly higher vascular flow than simple FAs [5]. In the present case, tumor size was 23 × 14 × 7 cm in a nonlactating and nonpregnant female. Out of the four essential features, sclerosing adenosis is the most common feature observed in complex FAs [3,7]. Complex FAs with two or more features have also been reported in the literature [2,3,7]. However, cases with all four features in the same patient are very few [2,3,7]. Complex FAs are reported to be associated with an increased risk of breast cancer (relative risk: 2.27-3.10) [3]. Interestingly, Sklair-Levy et al. observed a lower incidence of carcinoma breast in patients with complex FAs [7]. Nassar et al. observed that none of the four complex FA features are significantly associated with an elevated risk of breast carcinoma [3]. However, they stated that the presence of two or more features is associated with an increased risk of breast carcinoma compared to only one feature [3]. The present case showed all four features of complex FAs with areas of infarction. Mucinous carcinoma and malignant phyllodes were considered as the differential. However, on histopathological examination, features of mucinous carcinoma and malignant phyllodes were not seen. In most cases, FAs do not require surgery as they shrink and disappear over time [1,7,8]. If their size is large, surgical excision should be done [1,7,8]. Some authors recommend follow-up in FAs with atypia over excisional biopsy, stating that atypia in FA does not increase the risk for breast carcinoma [9]. There is limited literature on the management of complex FA with or without atypia [7]. Greenberg et al. recommended that all FAs with complex features be surgically removed soon after diagnosis [8]. However, Sklair-Levy et al. recommended similar conservative management of complex FAs as that of simple FAs as they observed low incidence of breast carcinoma in cases of FAs [7]. In the present case, the tumor was surgically managed due to its large size.

## Conclusions

Detailed clinical, radiological evaluation and comprehensive workup may help in early and breast-conserving management. Patients with giant FAs should undergo a regular radiological examination to diagnose any other lesion following giant FAs. The present case was unique due to its large size and all four pathognomonic features of complex FAs.
